# Dentin abrasivity of various desensitizing toothpastes

**DOI:** 10.1186/s13005-016-0113-1

**Published:** 2016-04-02

**Authors:** W. H. Arnold, Ch. Gröger, M. Bizhang, E. A. Naumova

**Affiliations:** Department of Biological and Material Sciences in Dentistry, School of Dentistry, Witten/Herdecke University, Witten, Germany; Department of Preventive and Operative Dentistry, School of Dentistry, Witten/Herdecke University, Witten, Germany

**Keywords:** Toothpaste, Dentin, Dentin tubules, Root dentin, Hypersensitivity

## Abstract

**Background:**

The aim of this study was to compare the abrasivity of various commercially available toothpastes that claim to reduce dentin hypersensitivity.

**Methods:**

Dentin discs were prepared from 70 human extracted molars. The discs were etched with lemon juice for 5 min, and one half of the discs were covered with aluminum tape. Following this, they were brushed with 6 different toothpastes, simulating a total brushing time of 6 months. As a negative control, discs were brushed with tap water only. The toothpastes contained pro-arginine and calcium carbonate, strontium acetate, stannous fluoride, zinc carbonate and hydroxyapatite, new silica, or tetrapotassium pyrophosphate and hydroxyapatite. After brushing, the height differences between the control halves and the brushed halves were determined with a profilometer and statistically compared using a Mann–Whitney U test for independent variables.

**Results:**

A significant difference (*p* < 0.001) in height difference between the controls and the toothpaste-treated samples was found in all cases, except for the stannous fluoride-containing toothpaste (*p* = 0.583). The highest abrasion was found in the toothpaste containing zinc carbonate and hydroxyapatite, and the lowest was found in the toothpaste containing pro-arginine and calcium carbonate.

**Conclusions:**

Desensitizing toothpastes with different desensitizing ingredients have different levels of abrasivity, which may have a negative effect on their desensitizing abilities over a long period of time.

## Background

The prevalence of dentin hypersensitivity has been increasing over the past decades [1], and there is a need for adequate treatment of this condition. The causes of dentin hypersensitivity include open dentin tubules due to gingival recession and subsequent cervical dentin erosion [2]. Dentin erosion occurs for a variety of reasons. Amongst them are erosive foods and beverages, as well as esophageal reflux or eating disorders [3]. Another reason for dentin erosion may be the use of toothpastes and toothbrushes [4]. Various strategies have been developed to handle this problem. They range from home-use dental products, such as desensitizing toothpastes [5–10], to in-office treatments, such as sealing dentin tubules either with a varnish [11–13] or with a dentin adhesive [14, 15]. The first choice treatment of dentin hypersensitivity is home-use dental products, mainly desensitizing toothpastes.

Desensitizing toothpastes are divided into two groups with different mechanisms of action. The first group comprises toothpastes that block pulp nerve responses, whereas the second group comprises toothpastes that occlude dentin tubules [16]. All desensitizing toothpastes have different ingredients, which have different effects on the ability to occlude dentin tubules [5]. All of these toothpastes are similar in that they have certain levels of abrasivity within a relative-dentin-abrasion (RDA) value range between 20 and 120. In a recent study, it was shown that toothpastes with high RDA values resulted in greater losses of dentin [4] after tooth brushing. The abrasivity of desensitizing toothpastes may have an adverse effect on the occlusion of dentin tubules because the tubules might be reopened during the brushing procedure.

Therefore, the aim of this study was to compare the abrasivity of various desensitizing toothpastes quantitatively. The null hypothesis stated that there is no difference in the abrasivity of the different included toothpastes.

## Methods

Seventy caries-free extracted human molars were used for this experimental study. The collection of the teeth was approved by the ethical committee of Witten/Herdecke University (116/2013). Informed verbal consent was obtained from the patients before the use of their teeth. The teeth were stored in 0.9 % NaCl containing 0.1 % thymol until use.

### Experimental design

From the 70 teeth, 3-mm-thick dentin discs were prepared using a saw microtome (Leica 1600, Leitz Wetzlar, Germany). The discs were randomly divided into 7 groups of 10 discs each and etched with lemon juice (Hitchcock, Mönchen Gladbach, Germany) for 5 min, and one half of each disc were covered with aluminum tape. Following this, the discs were placed into a tooth-brushing machine, and a tooth brushing time of 6 months was simulated. The brushing time was calculated as follows: 28 teeth per oral cavity assuming a vestibular and an oral surface = 56 surfaces. A recommended brushing time of 360 s per day results in a brushing time of 6.4 s per tooth surface. This is multiplied by 182.5 days (6 months) and results in a total brushing time per tooth surface of 19 min 33 s. The used toothbrush has an active brushing field of 28 mm length which would cover two tooth surfaces at one time in the oral cavity, therefore, the brushing time was again doubled and resulted in a total bushing time per surface of 39 min and 6 s. As toothbrush the American Dental Association Standard Toothbrush was used. The toothbrush load was 2 N. The standard toothbrush of the American Dental Association was used with 120 linear strokes per min. The toothpastes and the active ingredients that were used are summarized in Table 1. One group served as a negative control and was brushed with tap water only. After tooth brushing, the aluminum tape was removed, and the height differences between the covered halves and the brushed halves of the discs were determined using an optical profilometer (Infinite focus G3, Alicona, Germany). Twenty measurements per disc were made, and the mean value was calculated for each disc.

**Table 1:** Summary of toothpastes used

Product name	Active ingredient	RDA value^a^	Company
BioRepair (#1)	Zinc carbonate hydroxyapatite	69	Dr. K. Wolff, Bielefeld, Germany
Elmex Sensitive Professional (#2)	Pro-arginine, calcium carbonate	30	CP-GABA, Hamburg, Germany
Elmex (#3)	Amine fluoride	77	CP-GABA, Hamburg, Germany
Sensodyne Rapid (#4)	Strontium acetate	70	GlaxoSmithKline, Brentford, UK
Sensodyne Repair (#5)	Stannous fluoride	119	GlaxoSmithKline, Brentford, UK
Dontodent Sensitive (#6)	Tetrapotassium pyrophosphate, hydroxyapatite	20	DM Dogeriemarkt, Karlsruhe, Germany

^a^RDA values were obtained from the manufacturer

### Statistical analysis

Sample size calculation was carried out (Axum 7, Mathsoft, Cambridge, Massachusetts, USA) with data obtained in a preliminary experiment with a power of 0.8 and a significance level of α < 0.05, revealing a minimum number of 8 specimens per group. The mean values of the height differences were compared between the different toothpastes and the negative controls using a Wilcoxon-Mann–Whitney test for independent variables and post hoc Bonferroni adjustment, which resulted in a final p value of 0.0083. The correlation between abrasivity and RDA value was calculated with the nonparametric Spearman-Rho test. Descriptive statistics are presented as boxplots. All calculations were performed with SPSS (IBM Corporation, Armonk, NY, USA; Rel. 21) statistical software.

## Results

**Fig. 1:**
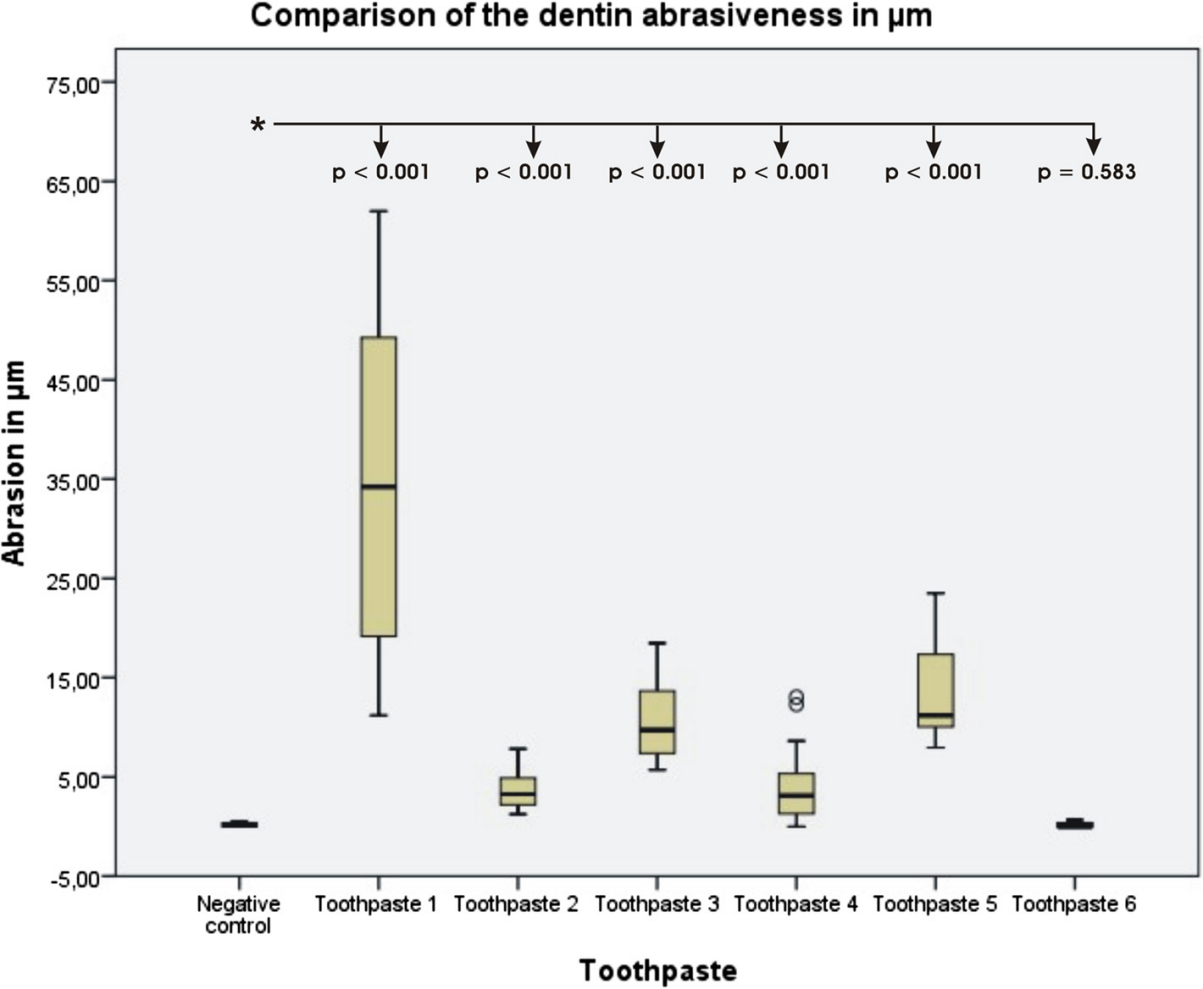
Boxplot graphics showing data distribution of abrasion values in μm. The box is representing 50 % of the measured values, whiskers the 75 % percentile and circles extremes, the box within the box marks the median

**Fig. 2:**
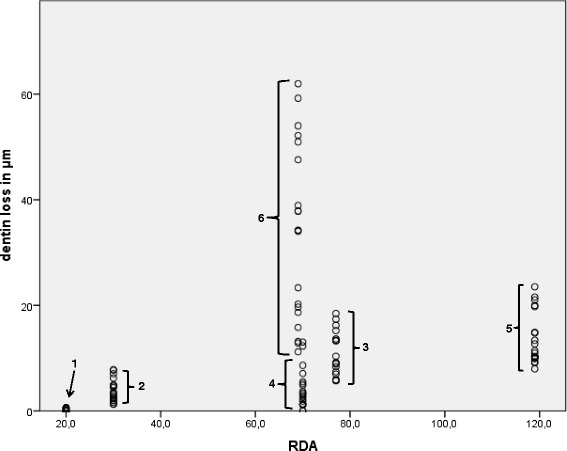
Correlation between dentin loss and RDA value of toothpaste. The numbers are representing the toothpaste numbers

The statistical evaluation showed significant differences (*p* < 0.001) between the negative control and toothpastes 1–5 (Fig. 1). The difference between toothpaste 6 with tetrapotassium pyrophosphate, hydroxyapatite and the negative control was not significant (*p* = 0.583). The exact descriptive data are summarized in Table 2. The highest abrasion was found in the toothpaste containing zinc carbonate and hydroxyapatite carbonate (toothpaste #1). A significant correlation (*p* < 0.001) between the height difference on the dentin discs and RDA value with a correlation coefficient of *r* = 0.568 was found. The graphic representation demonstrated a rather mild correlation (Fig. 2)

**Table 2:** Descriptive data of abrasion values

Toothpaste #	Median	Minimum	Maximum	Interquartile range
1	34.20	11.22	61.98	50.76
2	32.66	1.25	7.84	6.59
3	9.73	5.72	18.47	12.75
4	3.11	0.00	13.04	13.04
5	11.21	7.96	23.52	15.55
6	0.00	0.00	0.67	0.67
tape water	0.02	0.00	0.49	0.49

All values are in μm

## Discussion

Erosion of the tooth surface in the cervical area results in a loss of covering cementum and an opening of dentin tubules, which in turn leads to dentin hypersensitivity. The use of desensitizing toothpastes is always the first recommendation for the treatment of dentin hypersensitivity [6]. The mechanism of action of the majority of desensitizing toothpastes is an effect on dentin tubule occlusion [5, 16]. The cleaning effect of toothpastes is due to their RDA values and other abrasive components such as nanoparticles. A high RDA value results in a large amount of dentin abrasion, which then might reopen occluded tubules and diminish the sensitizing effect.

The results of this study demonstrated large differences in the abrasivity of the various investigated desensitizing toothpastes. The abrasivity of toothpaste is dependent on numerous factors. The main factor is the content of abrasives [17]. All toothpastes used in this study employed silica as an abrasive substance. However, other ingredients, such as CaCO_3,_ hydroxyapatite, and other nanoparticles, may also contribute to the abrasivity of toothpaste [17]. All toothpastes contain a variety of different ingredients, which makes it almost impossible to determine the influence of a certain substance on the abrasivity of a toothpaste. This is emphasized by the results of this investigation. Especially toothpaste 6 demonstrated no abrasiveness compared to water but does contain tetrapotassium pyrophosphate, hydroxyapatite as active ingredient. It remains speculative weather the hydroxyapatite particles are too small for being abrasive. The influence of the RDA on the loss of hard dental tissue has been discussed widely in the literature [4, 17–20]. In this study, a correlation between RDA value and amount of dentin loss was also found. Although the correlation was significant (*p* < 0.001), the correlation coefficient was not very strong (*r* = 0.568), and the graphic representation did not show a clear linear correlation between increasing RDA value and amount of substance loss. This finding is in accordance with the results of another study, which did not find a correlation between RDA value and dentin abrasivity [21]. The reason for this mild correlation might be the relatively low number of investigated specimens.

There is still an ongoing debate as to whether toothpastes are contributing to dentine hypersensitivity [18, 22]. Desensitizing toothpastes should remove the smear layer on dentin and leave deposits of particles, which occlude dentin tubules [2, 23]. Another study showed that desensitizing toothpastes partly occlude dentin tubules [5], but the abrasivity of toothpastes was not investigated. In this study, it could be shown that under experimental conditions and after dentin erosion a maximum of 61.98 μm dentin was removed. Therefore, it is very likely that dentine tubules were not occluded in these cases.

## Conclusions

Within the limitations of an in vitro study, it can be concluded that different desensitizing toothpastes have different abrasivity regardless of their content of active desensitizing ingredients. Abrasivity of toothpastes may hamper their desensitizing effects.
